# Maternal caffeine intake during pregnancy and risk of fetal growth restriction: a large prospective observational study

**DOI:** 10.1136/bmj.a2332

**Published:** 2008-11-03

**Authors:** CARE Study Group

## Abstract

**Objective** To examine the association of maternal caffeine intake with fetal growth restriction.

**Design** Prospective longitudinal observational study.

**Setting** Two large UK hospital maternity units.

**Participants** 2635 low risk pregnant women recruited between 8-12 weeks of pregnancy.

**Investigations** Quantification of total caffeine intake from 4 weeks before conception and throughout pregnancy was undertaken with a validated caffeine assessment tool. Caffeine half life (proxy for clearance) was determined by measuring caffeine in saliva after a caffeine challenge. Smoking and alcohol were assessed by self reported status and by measuring salivary cotinine concentrations.

**Main outcome measures** Fetal growth restriction, as defined by customised birth weight centile, adjusted for alcohol intake and salivary cotinine concentrations.

**Results** Caffeine consumption throughout pregnancy was associated with an increased risk of fetal growth restriction (odds ratios 1.2 (95% CI 0.9 to 1.6) for 100-199 mg/day, 1.5 (1.1 to 2.1) for 200-299 mg/day, and 1.4 (1.0 to 2.0) for >300 mg/day compared with <100 mg/day; test for trend P<0.001). Mean caffeine consumption decreased in the first trimester and increased in the third. The association between caffeine and fetal growth restriction was stronger in women with a faster compared to a slower caffeine clearance (test for interaction, P=0.06).

**Conclusions** Caffeine consumption during pregnancy was associated with an increased risk of fetal growth restriction and this association continued throughout pregnancy. Sensible advice would be to reduce caffeine intake before conception and throughout pregnancy.

## Introduction

Caffeine is the most widely consumed xenobiotic in pregnancy, with the potential to adversely affect the developing fetoplacental unit. Maternal caffeine intake has been reported to be associated with a reduction in birth weight,^1^ ^2^ ^3^ ^4^ ^5^ but the precise level of intake above which the risk is increased remains unknown. Caffeine intake of ≥300 mg/day has been associated with fetal growth restriction,^6^ ^7^ ^8^ but Vlajinac et al found a significant reduction in infant birth weight of 114 g with maternal caffeine consumption of as little as 141 mg/day.^9^ More controversially, others have shown that maternal caffeine concentration has an inverse association with birth weight when confounders such as smoking were taken into account.^2^ ^10^ ^11^ In 2001 the Committee on Toxicity of Chemicals in Food, UK, after a thorough review of the literature, concluded that, although caffeine intake >300 mg/day might be associated with low birth weight and spontaneous miscarriage, the evidence was inconclusive.^12^

Possible reasons for these inconsistent outcomes include inaccurate estimation of caffeine consumption, including an assumption that tea and coffee are the only sources of caffeine,^3^ ^9^ ^10^ retrospective assessment of caffeine intake,^2^ ^10^ ^13^ ^14^ ^15^ assessment of association based on consumption in individual trimesters rather than throughout pregnancy,^4^ ^9^ ^10^ ^13^ failure to allow for individual variations in caffeine metabolism,^4^ ^16^ inadequate control for confounding factors such as smoking and alcohol consumption,^17^ ^18^ and non-uniformity in defining the primary outcome measures.^1^ ^2^ ^4^ ^6^ ^9^ ^10^ ^15^ ^16^

Caffeine is rapidly absorbed and crosses the placenta freely.^19^ After ingestion of 200 mg caffeine, intervillous blood flow in the placenta was found to be reduced by 25%.^20^ Cytochrome P450 1A2, the principal enzyme involved in caffeine metabolism, is absent in the placenta and the fetus.^21^ The amount of caffeine and metabolites available to the fetoplacental unit therefore depends on the maternal caffeine metabolism, which shows marked variation between individuals because of genetic and environmental factors such as nicotine.^22^ ^23^ ^24^ Variations in caffeine metabolic activity have been found to be more closely associated with fetal growth restriction than have blood caffeine concentrations.^25^ Therefore, any comprehensive study of the effects of caffeine on fetal growth must include an assessment of caffeine metabolism.

In order to examine the association of maternal caffeine intake on fetal growth, we used a validated, robust caffeine assessment tool to quantify total caffeine intake, from all possible sources, throughout pregnancy.^26^ Using these data, and taking into account individual variation in caffeine metabolism, we aimed to establish the safe upper limit of caffeine consumption with respect to adverse pregnancy outcome (specifically fetal growth restriction).

## Methods

### Participants

We prospectively recruited low risk pregnant women from two large UK teaching hospital maternity units (Leeds and Leicester) from September 2003 to June 2006. The inclusion criteria included age 18-45 years and singleton pregnancies accurately dated by ultrasound. Women with concurrent medical disorders, psychiatric illness, HIV infection, or hepatitis B infection were excluded. We identified eligible women by screening their pre-booking maternity notes, then sent them detailed information about the study and asked them to return a reply slip about their willingness to take part in the study. Personal contacts were then made with those who agreed to participate. This initial visit was conducted at the hospital or at the volunteer’s general practice or home by a clinical research fellow (Leicester) or a midwife (Leicester and Leeds) at 8-12 weeks gestation. Volunteers’ demographic details (age, parity, maternal height, weight, socioeconomic status, and gestational age) were recorded by means of a questionnaire.

### Quantification of caffeine intake

Caffeine intake was estimated with a validated caffeine assessment tool, a questionnaire designed at the University of Leeds, to record habitual caffeine intake before and during pregnancy.^26^ Information in the questionnaire included estimates of caffeine content from all potential dietary sources and over the counter drugs and details of potential confounders such as smoking, alcohol intake, and nausea. We recorded specific brand names, portion sizes, methods of preparation, and quantity and frequency of intake for different gestational periods. We also obtained details of caffeine content for each item from published reports,^27^ manufacturers, and coffee houses, and, from these, we estimated precise caffeine intakes using an SPSSv14 program developed in-house.^26^ Three caffeine assessment tools were administered by the clinical research fellow and research midwives to determine caffeine intake in pregnancy—the first, administered at recruitment by the researcher, included aspects of recall of caffeine intake from four weeks before pregnancy until recruitment into the study at 8-12 weeks of pregnancy; the second covered the period 13-28 weeks; and the third included the period 29-40 weeks of pregnancy.

### Saliva sample collection, storage, and transport

Saliva samples for determining nicotine exposure (defined as baseline values before the caffeine challenge) were collected from women at recruitment, using a Salivette (Sarstedt, Aktiengesellschaft, Loughborough, UK) kept in the mouth for 5-10 minutes. Additionally, we assessed caffeine half life from a caffeine challenge test (adapted from Butler et al^28^) performed within two weeks of recruitment. We provided participants with appropriate materials and instructions to perform the test at home, and the samples were then returned in a prepaid envelope. The test involved overnight fasting, followed by the challenge (a drink of 500 ml diet cola containing 63.5 mg caffeine ingested over a period of 20 minutes) with no other caffeine consumed during the challenge. Participants then collected saliva samples about one and five hours after the challenge. Precise sample collection times and details of drinks or food consumed during the test period were recorded on a questionnaire. When samples arrived at the laboratory, saliva was isolated from the Salivettes by centrifugation and stored at −80°C.

### Biochemical analyses

All samples were analysed in the Molecular Epidemiology Unit (University of Leeds).

**Salivary caffeine—**Salivary caffeine was extracted and quantified using liquid:liquid extraction and reversed phase high performance liquid chromatography (HPLC) with ultraviolet detection.^26^ We calculated the half life for caffeine from salivary caffeine concentrations recorded at one and five hours after the caffeine challenge.

**Salivary cotinine—**Salivary cotinine concentrations in samples taken at recruitment were quantified by means of enzyme linked immunosorbent assay (ELISA) (Cozart Bioscience, Oxfordshire, UK) according to the manufacturer’s instructions. We then classified participants on the basis of these cotinine concentrations as active smokers (>5 ng/ml), passive smokers (1-5 ng/ml), or non-smokers (<1 ng/ml).^29^

### Pregnancy outcomes

We obtained information on antenatal pregnancy complications and delivery details (gestational age at delivery, birth weight, and sex of the baby) from the electronic maternity databases.

The primary outcome measure was fetal growth restriction defined as birth weight <10th centile on a customised centile chart which takes into account maternal height, weight, ethnicity, and parity and neonatal birth weight and sex (www.gestation.net).^30^ We chose this definition as it is the most commonly used and because, although not all those cases classified as fetal growth restriction would be pathological, it is likely to include most pathological fetal growth restrictions. In addition, we assessed the association of maternal caffeine intake with birth weight. 

Other pregnancy outcomes studied were late miscarriage (spontaneous pregnancy loss between 12 and 24 weeks), preterm delivery (delivery at <37 completed weeks), gestational hypertension (blood pressure ≥140/90 mmHg on more than one occasion 4 hours apart after >20 weeks of pregnancy), proteinuric hypertension (gestational hypertension and proteinuria of ≥300 mg protein in 24 hours, based on the International Society for the Study of Hypertension in Pregnancy^31^), and stillbirth (delivery ≥24 weeks with no signs of life at birth).

### Statistical methods

We expressed participants’ caffeine consumption in mg/day averaged over the whole pregnancy and for the individual trimesters. To estimate the sample size required, we assumed that the mean caffeine intake during pregnancy was 206 mg/day,^4^ and that caffeine followed a log normal distribution, with a coefficient of variation of 1. Assuming that 10% of births showed fetal growth restriction, then 3000 births would give 80% power to detect a difference of 30 mg/day in caffeine intakes between mothers of babies with restricted fetal growth and mothers of babies of appropriate weight for gestational age with type I error set at 0.05. This also gave 80% power to detect an odds ratio for fetal growth restriction of 1.4 between high and low caffeine consumers (defined as being above or below the median caffeine intake).

We performed unconditional logistic regression modelling for fetal growth restriction and general linear modelling for birth weight, with stratification for the two maternity units, using Stata version 10 survey facilities.^32^ Maternal height, weight, ethnicity, and parity at booking and neonatal gestation at delivery and sex were taken into account in the definition for fetal growth restriction, and were adjusted for in the model for birth weight. We also made statistical adjustment for salivary cotinine levels and self reported alcohol consumption in all models. We conducted sensitivity analyses to assess the robustness of the results to adjustment for nausea, exclusion of high risk pregnancies, multiparity, extremely high or low caffeine intakes, and the maternity unit.

We also assessed the relation between the risk of fetal growth restriction and maternal caffeine intake during pregnancy by considering caffeine intake as a continuous variable: after adjusting for the factors mentioned above, we performed modelling using the best fitting, second order, fractional polynomial with 95% confidence intervals.

Caffeine half life as assessed by the caffeine challenge test was not normally distributed. We therefore categorised women in relation to the median value as having a shorter half life (faster caffeine clearance from the circulation) or longer half life (slower clearance). We stratified the odds ratio for fetal growth restriction by caffeine half life (as a proxy for clearance) and intake after taking account of maternal age, weight, height, ethnicity, and parity and neonatal gestation and sex and adjusting for smoking status, amount smoked (cotinine concentration), and alcohol intake.

## Results

Over a period of three years, 13 071 eligible women were invited to participate from the two maternity units, and 2635 (20%) consented. Table 1 shows the demographic and clinical characteristics of the study population. The prevalence of fetal growth restriction in the cohort was 343/2635 (13%). The mean alcohol intake during pregnancy was 0.4 (95% confidence interval 0 to 9) units/day, with the highest consumption occurring, as might be expected, before conception and during the first four weeks of pregnancy.

**Table 1 tbl1:** Demographic and clinical characteristics of 2635 pregnant women and their babies, according to pregnancy outcome. Values are numbers (percentages) unless stated otherwise

	Pregnancy outcome		
	Fetal growth restriction (n=343)	Appropriate fetal growth (n=2292)	Total (n=2635)
**Maternal characteristics**			
Mean (SD) age (years)	30.0 (6.6)	29.8 (6.5)	30 (6.6)
Mean (SD) weight before pregnancy (kg)	66.7 (13.2)	66.8 (12.6)	66.8 (13.1)
Mean (SD) body mass index before pregnancy (kg/m^2^)	24.5 (4.5)	24.5 (4.6)	24.5 (4.5)
Primiparous	186 (55)	1042 (46)	1228 (47)
Preterm labour	29 (8)	77 (3)	106 (4)
Gestational hypertension or pre-eclampsia	25 (7)	42 (2)	67 (3)
Stillbirth	3 (0.9)	6 (0.3)	9 (0.3)
Late miscarriage	3 (0.9)	16 (0.7)	19 (0.7)
**Neonatal characteristics**			
Mean (SD) gestational age at delivery (weeks)	40 (3)	40 (2)	40 (2)
Mean (SD) birth weight (g)	2750 (520)	3560 (470)	3450 (550)
Male	172 (50)	1152 (52)	1324 (51)

### Caffeine intake during pregnancy

The women’s mean caffeine intake during pregnancy was 159 mg/day (table 2). It decreased from 238 mg/day before pregnancy to 139 mg/day between weeks 5 and 12 of pregnancy and remained at about this level until the third trimester, when it gradually increased to 153 mg/day. About 62% of the caffeine ingested by the women during pregnancy was from tea. Other important sources were coffee (14%), cola drinks (12%), chocolate (8%), and soft drinks (2%). Hot chocolate, energy drinks, and alcoholic drinks contributed 2%, 1%, and <1% respectively. Over the counter drugs made a negligible contribution to the total caffeine intake.

**Table 2 tbl2:** Mean caffeine and alcohol intake and smoking status among 2635 pregnant women according to pregnancy outcome. Values are numbers (percentages) unless stated otherwise

Characteristic	Pregnancy outcome		
	Fetal growth restriction (n=343)	Appropriate fetal growth (n=2292)	Total (n=2635)
Mean (SD) caffeine intake (mg/day):			
Throughout pregnancy	200 (202)	153 (145)	159 (154)
First trimester	201 (206)	157 (160)	163 (167)
Second trimester	184 (207)	141 (144)	147 (156)
Third trimester	197 (222)	143 (146)	153 (164)
Caffeine intake during pregnancy:			
<100 mg/day	122 (36)	1000 (46)	1122 (44)
100-199 mg/day	90 (27)	601 (27)	691 (27)
200-299 mg/day	63 (19)	313 (14)	376 (15)
≥300 mg/day	63 (19)	284 (13)	347 (14)
Mean (SD) alcohol intake (units/day):			
Throughout pregnancy	0.4 (0.7)	0.4 (0.5)	0.4 (0.6)
First trimester	0.6 (0.9)	0.4 (0.7)	0.5 (0.8)
Second trimester	0.2 (0.4)	0.2 (0.5)	0.2 (0.5)
Third trimester	0.3(0.4)	0.2 (0.5)	0.3 (0.5)
Smoking status (n=2509)*:			
Non-smoker	213 (64)	1622 (75)	1835 (73)
Passive smoker	39 (12)	268 (12)	307 (12)
Current smoker	79 (24)	288 (13)	367 (15)

*Smoking status based on salivary cotinine concentrations: non-smoker <1 ng/ml, passive smoker 1-5 ng/ml, current smoker >5 ng/ml.

### Relation between caffeine intake in pregnancy and fetal growth

The relation between total caffeine intake in pregnancy and fetal growth restriction showed a significant trend with increasing caffeine intake (test for trend P=0.02, table 3). Compared with caffeine intake of <100 mg/day, the odds ratio of having a growth restricted baby increased to 1.2 (95% confidence interval 0.9 to 1.6) for intakes of 100-199 mg/day, to 1.5 (1.1 to 2.1) for intakes of 200-299 mg/day, and to 1.4 (1.0 to 2.0) for intakes of ≥300 mg/day. This relation was consistent across all three trimesters.

**Table 3 tbl3:** Risk of fetal growth restriction among offspring of 2635 pregnant women according to caffeine intake during pregnancy

Caffeine intake (mg/day)	Unadjusted risk*			Adjusted risk†	
	Odds ratio (95% CI)	Test for trend		Odds ratio (95% CI)	Test for trend
Average over pregnancy:					
<100	1	P<0.001		1	P=0.02
100-199	1.2 (0.9 to 1.6)			1.2 (0.9 to 1.6)	
200-299	1.6 (1.2 to 2.3)			1.5 (1.1 to 2.1)	
≥300	1.8 (1.3 to 2.5)			1.4 (1.0 to 2.0)	
In weeks 5-12:					
<100	1	P<0.001		1	P=0.05
100-199	1.2 (0.9 to 1.6)			1.1 (0.8 to 1.5)	
200-299	1.4 (1.0 to 2.0)			1.3 (0.9 to 1.9)	
≥300	1.8 (1.3 to 2.5)			1.4 (1.0 to 1.9)	
In weeks 13-28:					
<100	1	P=0.001		1	P=0.02
100-199	1.5 (1.1 to 2.0)			1.4 (1.0 to 2.0)	
200-299	1.8 (1.3 to 2.6)			1.7 (1.2 to 2.4)	
≥300	1.6 (1.1 to 2.4)			1.3 (0.9 to 2.0)	
In weeks 29-40:					
<100	1	P<0.001		1	P=0.004
100-199	1.4 (1.0 to 1.9)			1.4 (1.0 to 2.0)	
200-299	1.9 (1.3 to 2.8)			1.8 (1.2 to 2.7)	
≥300	1.9 (1.3 to 2.8)			1.6 (1.0 to 2.4)	

*Unadjusted odds ratios take account of maternal age, weight, height, ethnicity, and parity and neonatal gestational age at delivery and sex.

†Adjusted odds ratios are also adjusted for smoking status (salivary cotinine concentration) and alcohol intake.

Caffeine consumption of >200 mg/day during pregnancy was associated with a reduction in birth weight of about 60-70 g, with a significant trend for greater reduction in birth weight with higher caffeine intake (P=0.004). This relation was consistent across all three trimesters (table 4).

**Table 4 tbl4:** Unadjusted and adjusted linear regression for birth weight among offspring of 2635 pregnant women according to caffeine intake during pregnancy

Caffeine intake (mg/day)	Unadjusted change in birth weight (g)			Adjusted change in birth weight (g)*	
	Change (95% CI)	Test for trend		Change (95% CI)	Test for trend
Average over pregnancy:					
<100	0	P<0.001		0	P=0.004
100-199	−1 (−51 to 50)			−21 (−62 to 20)	
200-299	−63 (−129 to 4)			−70 (−123 to −18)	
≥300	−144 (−221 to −66)			−63 (−119 to −6)	
In weeks 5-12:					
<100	0	P<0.001		0	P=0.009
100-199	−6 (−58 to 45)			−34 (−76 to 8)	
200-299	−66 (−134 to 2)			−61 (−112 to −9)	
≥300	−144 (−220 to −69)			−59 (−114 to −4)	
In weeks 13-28:					
<100	0	P=0.003		0	P=0.006
100-199	−15 (−74 to 44)			−24 (−72 to 24)	
200-299	−44 (−119 to 30)			−65 (−124 to −6)	
≥300	−129 (−212 to 46)			−74 (−138 to −10)	
In weeks 29-40:					
<100	0	P=0.009		0	P=0.004
100-199	−25 (−98 to 48)			−66 (−125 to −7)	
200-299	−61 (−154 to 31)			−69 (−141 to 3)	
≥300	−119 (−211 to −27)			−89 (−158 to −21)	

*Adjusted estimates take account of maternal age, weight, height, ethnicity, parity, smoking status (salivary cotinine concentration), and alcohol intake and neonatal gestational age at delivery and sex.

In a small cohort of women (n=109) who had reduced their caffeine intake from 300 mg/day before pregnancy to <50 mg/day by weeks 5-12 of pregnancy their offspring’s mean birth weight was higher than that of those who maintained their caffeine intake at >300 mg/day (n=193) (difference in birth weight 161 g (95% confidence interval 24 to 297 g), P=0.02).

To examine possible threshold effects, we analysed the relation between the estimated risk of delivering a growth restricted fetus and maternal caffeine intake during pregnancy measured as a continuous variable (fig 1). There was a rapid increase in associated risk from increasing caffeine intake up to about 30 mg/day. Thereafter, estimated risk continued to rise roughly linearly in a dose-response relation. At no point did the estimated risk cease to increase with increasing caffeine intake. There was no observed plateau effect.

**Figure fig1:**
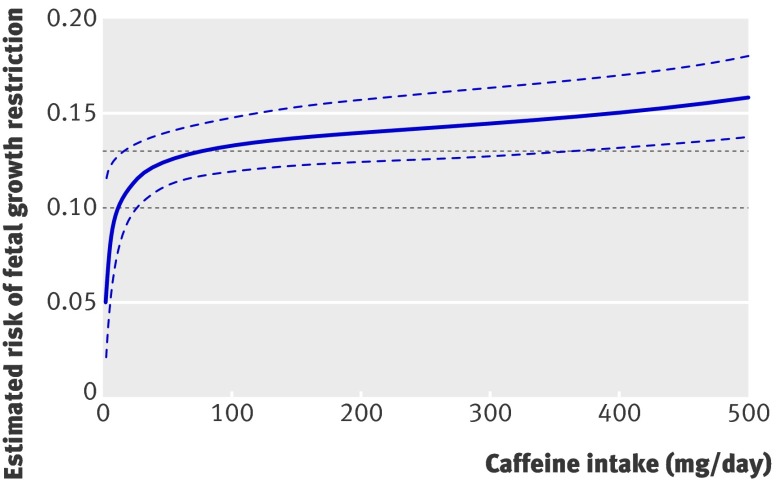
**Fig 1** Relation between risk of fetal growth restriction and caffeine intake (mg/day) during pregnancy. The relation is modelled by the best-fitting second-order fractional polynomial, with 95% confidence intervals. The graph is restricted to <500 mg/day for clarity. Horizontal dotted lines mark national average risk of fetal growth restriction (10%) and average risk in study cohort (13%)

### Relation between caffeine clearance and fetal growth

Using maternal caffeine half life as a proxy for clearance rate, we found some evidence that the association between caffeine intake and fetal growth restriction was stronger in women with a faster caffeine clearance than in those with slower clearance (test for interaction, P=0.06) (table 5).

**Table 5 tbl5:** Stratification of risk of fetal growth restriction among offspring of 2635 pregnant women according to caffeine intake during pregnancy and caffeine half life (proxy for clearance)

Caffeine intake (mg/day)	Risk of fetal growth restriction*	
	Odds ratio (95% CI)	Test for trend
**Shorter caffeine half life (n=774)†**		
<100	1	P=0.02
100-199	1.6 (0.9 to 3.0)	
200-299	2.4 (1.3 to 4.4)	
≥300	1.7 (0.9 to 3.3)	
**Longer caffeine half life (n=764)†**		
<100	1	P=0.8
100-199	1.1 (0.6 to 1.7)	
200-299	0.6 (0.3 to1.3)	
≥300	1.5 (0.7 to 2.9)	
	Test for interaction of half life P=0.06	

*Adjusted for maternal age, weight, height, ethnicity, parity, smoking status (salivary cotinine concentration), and alcohol intake and neonatal gestational age at delivery and sex.

†Shorter caffeine half life (≤median value)=faster clearance; longer half life (>median value)=slower clearance).

### Relation between smoking in pregnancy and fetal growth

Women classified as active smokers (based on their salivary cotinine concentrations) had nearly twice the risk of fetal growth restriction compared with women classified as non-smokers (adjusted odds ratio 1.9 (95% confidence interval 1.4 to 2.6), P<0.001). The birth weights of babies born to active smokers were 178 g lighter (95% confidence interval 127 to 230 g) than those born to non-smokers (P<0.001). Adjusting for nausea (reported by 81% of the population in the first trimester) did not alter these results.

## Discussion

This is one of the largest prospective studies investigating the association of maternal caffeine intake with fetal growth. Maternal caffeine intake was associated with an increased risk of fetal growth restriction even after adjustment for smoking and alcohol intake. We could find no level of intake at which there was no association with increased risk of fetal growth restriction. The size of the association for caffeine was of a similar size to that for alcohol intake in pregnant women in this study (data not shown).

The strong association between caffeine intake and birth weight was maintained across all of the trimesters. However, from these results we cannot define a critical time window for any maximal effect. This clearly warrants further investigation.

### Strengths and weaknesses of the study

Although only 20% of the women we invited took part in the study, this low response rate does not lessen the validity of our data, as the association of caffeine with birth weight should not be different from that in the general population, especially as various confounders were taken into consideration. In addition, examination of our maternity databases indicated that the population we studied was similar to that of the maternity units as a whole.

A major strength of our study is that we have objectively quantified caffeine from all known sources. Caffeine intake was validated by comparison with a food diary and repeated measures of exposure from saliva samples,^27^ and we believe that, for the first time, this reflects a true picture of total caffeine intake by women during pregnancy. More than 60% of the caffeine consumed was from tea, and only 14% from coffee. Our findings emphasise the weaknesses of studies where caffeine intake was equated to that of coffee alone. Weng et al reported that coffee was the sole source of caffeine in 19% of their pregnant cohort, and 44% consumed caffeine from combined caffeine and non-caffeine sources.^33^ Since 26% of caffeine intake in our cohort was from neither coffee nor tea, studies that concentrated on coffee and tea alone would have grossly underestimated caffeine intake.

### Study results in comparison with other studies 

Caffeine consumption almost halved in early pregnancy (from 250 mg/day before pregnancy to 150 mg/day in the first trimester), as has been reported elsewhere.^34^ The mean caffeine intake throughout pregnancy was much lower than the limit of 300 mg/day recommended by the UK government’s Food Standards Agency^12^ and in the USA.^35^

Several studies have concluded that caffeine intake of >300 mg/day is associated with low birth weight or fetal growth restriction.^6^ ^7^ ^8^ Our study confirms these findings and further defines the nature of the association. We could find no level of intake at which there was no association with increased risk of fetal growth restriction, and this risk was maintained throughout pregnancy. Although the overall size of the reduction in birth weight may be seen as small, an extra 60-70 g in weight could reduce perinatal morbidity and mortality in an already compromised fetus. The steep decline in risk associated with caffeine intakes of <30 mg/day may be attributable to unmeasured confounding. Furthermore, women who consume little or no caffeine may be generally more health conscious than those who consume more, and the effect may be one for which we have been unable to adjust.

We found that average caffeine consumption of >100 mg/day was associated with a reduction in birth weight of 34-59 g in the first trimester, 24-74 g in the second, and 66-89 g in the third (after adjustment for smoking status and alcohol intake). Similar results were seen by Bracken et al in a prospective study of 2291 pregnant women in the US, where mean birth weight was reduced by 28 g for every 100 mg/day of caffeine consumed (P=0.0001), but the risk for fetal growth restriction was unchanged (odds ratio 0.96).^36^ This difference could be explained by methodological differences in the studies. 

A Danish cohort of 1207 women drinking at least three cups of coffee a day before 20 weeks of pregnancy were randomised to receive either caffeinated or decaffeinated instant coffee: there was no significant difference in birth weight between the two groups after adjustment for parity, gestational age at birth, and smoking.^37^ However, these women were recruited in the second half of pregnancy, so the effect of first trimester caffeine intake was not assessed, and there was no biochemical confirmation of participants’ compliance with caffeinated or decaffeinated coffee consumption. 

In addition, Bicalho and Filho reported no association between maternal caffeine consumption and low birth weight after adjusting for confounding variables in a case-control study in Brazil.^38^

### Caffeine metabolism

Some of the variation in previously reported associations between caffeine intake and pregnancy outcomes may reflect the effect of differences in caffeine metabolism. The degree to which a fetus is exposed to caffeine and its metabolites, which pass freely across the placenta, depends on maternal cytochrome P450 1A2 (CYP1A2) activity because this enzyme is absent in the fetus. We complemented our assessment of caffeine intake with a measure of caffeine metabolism and observed that the association of caffeine intake with fetal growth restriction was greater among women with faster caffeine clearance. 

Caffeine is primarily metabolised in the human liver to paraxanthine,^39^ but there is little data about metabolism in pregnant women. In our study caffeine was metabolised to paraxanthine, theobromine, and theophylline, with theobromine present in highest concentration in most of the women. As we were unable to measure the rate of formation or subsequent metabolism of these primary metabolites, we cannot attribute the association with fetal growth to any single metabolite. The association we observed may be due to caffeine itself or one of its metabolites, or to any combination of them. 

In a study of pregnant women who smoked, Klebanoff et al reported a positive association between maternal paraxanthine concentration in the third trimester and having an infant that was small for its gestational age.^40^ In another study, the highest concentrations of paraxanthine were associated with an increased risk of spontaneous abortion.^41^ Recently, higher cord blood paraxanthine concentrations have been shown to be associated with an increased risk of intrauterine growth restriction after adjustment for caffeine levels, implying an effect of CYP1A2 activity rather than absolute levels of paraxanthine.^25^ Further consideration of the role of CYP1A2 activity and caffeine metabolites is clearly warranted.

### Conclusion

This large prospective cohort study has demonstrated that maternal caffeine intake is associated with an increased risk of fetal growth restriction. The threshold at which this risk is significantly higher is not well characterised, but our data confirm that the association of fetal growth restriction with caffeine is reduced for those consuming <100 mg/day. We suggest that sensible advice for women contemplating pregnancy is to reduce their caffeine intake from all sources before conception. Once pregnancy is confirmed, they should make every effort to stop or markedly reduce caffeine consumption.

**What is already known on this topic**

Caffeine is the most common xenobiotic consumed in pregnancy, and there are conflicting results regarding the association of increased caffeine intake in pregnancy with fetal growth restriction and low birth weight
These differences could be explained by inconsistencies in accurate quantification of caffeine and in the definition of fetal growth restriction

**What this study adds**

Maternal caffeine intake is associated with an increased risk of fetal growth restriction after adjustment for smoking and alcohol intake
The size of the association for caffeine intake with fetal growth restriction is similar to that for alcohol intake
The association of caffeine with fetal growth restriction seems to be stronger in women with faster caffeine clearance
Sensible advice to pregnant women would be to reduce caffeine intake before conception and during pregnancy

## Notes

We thank Gordon Gibson, Fred Kadlubar, and Mark Klebanoff for their useful comments during the study. The Leicester team of the CARE Study Group thank Vilas Misty, Clare Lawrence, Bhavin Daudia, and the Department of Chemical Pathology, University Hospitals of Leicester NHS Trust, for sample handling and processing.

Members of the CARE Study Group:

Leeds team: Sinead Boylan, Janet E Cade, Vivien A Dolby, Darren C Greenwood, Alastair W M Hay, Sara F L Kirk, Susan Shires, Nigel Simpson, James D Thomas, James Walker, Kay L M White, Christopher P Wild, Centre for Epidemiology and Biostatistics, University of Leeds, Leeds LS2 9JT

Leicester team: Neelam Potdar, Justin C Konje, Nicholas Taub, Jim Charvill, Karen C Chipps, Shabira Kassam, Chetan Ghandi, , Marcus S Cooke, Departments of Cancer Studies and Molecular Medicine and Health Sciences, University of Leicester, Leicester LE2 7LX

Steering group: Justin C Konje (chair), Marcus Cooke (principal investigator), Leicester; Janet Cade (principal investigator), Leeds; David Gott, Natalie Thatcher, Stuart Creton, Caroline Tahourdin, Food Standards Agency, London; Gordon Gibson, University of Surrey

Statisticians: Darren Greenwood, Leeds; Nicholas Taub, Leicester; Clifton Gay, Food Standards Agency

Clinicians: Neelam Potdar, Justin C Konje, Leicester; Nigel Simpson, James Walker, Leeds

Research midwives: Viv Dolby, Heather Ong, Leeds; Shabira Kassam, Karen Chipps, Leicester

Nutritional methods: Sinead Boyland, Sara Kirk, Janet Cade, Leeds

Laboratory methods: Kay White, Susan Shires, Alastair Hay, Christopher Wild, Leeds; Marcus Cooke, Leicester

Database management: James Thomas, Ellen Hill, nutritionist students, Leeds; Jim Charvill, Chetan Ghandi, Leicester.

Ethical approval: Obtained from the local ethics committees, Directorate of Research and Development, Leicester and Leeds, LREC Ref 7260. Participants gave signed informed consent before enrolment into the study.

## Notes

**Cite this as: ***BMJ* 2008;337:a2332

## References

[1] Mau G, Netter P. Are coffee and alcohol consumption risk factors in pregnancy? [author’s translation]. Geburtshilfe Frauenheilkd 1974;34:1018-22. 4457384

[2] Beaulac-Baillargeon L, Desrosiers C. Caffeine-cigarette interaction on fetal growth. Am J Obstet Gynecol 1987;157:1236-40. 3688080 10.1016/s0002-9378(87)80301-0

[3] Fortier I, Marcoux S, Beaulac-Baillargeon L. Relation of caffeine intake during pregnancy to intrauterine growth retardation and preterm birth [see comment]. Am J Epidemiol 1993;137:931-40. 8317450 10.1093/oxfordjournals.aje.a116763

[4] Vik T, Bakketeig LS, Trygg KU, Lund-Larsen K, Jacobsen G. High caffeine consumption in the third trimester of pregnancy: gender-specific effects on fetal growth. Paediatr Perinat Epidemiol 2003;17:324-31. 14629313 10.1046/j.1365-3016.2003.00507.x

[5] Eskenazi B, Stapleton AL, Kharrazi M, Chee WY, Eskenazi B, Stapleton AL, et al. Associations between maternal decaffeinated and caffeinated coffee consumption and fetal growth and gestational duration. Epidemiology 1999;10:242-9. 10230832

[6] Martin TR, Bracken MB. The association between low birth weight and caffeine consumption during pregnancy. Am J Epidemiol 1987;126:813-21. 3661529 10.1093/oxfordjournals.aje.a114718

[7] Fenster L, Eskenazi B, Windham GC, Swan SH. Caffeine consumption during pregnancy and fetal growth. Am J Public Health 1991;81:458-61. 2003624 10.2105/ajph.81.4.458 PMC1405033

[8] Peacock JL, Bland JM, Anderson HR. Effects on birthweight of alcohol and caffeine consumption in smoking women. J Epidemiol Community Health 1991;45:159-63. 2072076 10.1136/jech.45.2.159 PMC1060735

[9] Vlajinac HD, Petrovic RR, Marinkovic JM, Sipetic SB, Adanja BJ. Effect of caffeine intake during pregnancy on birth weight. Am J Epidemiol 1997;145:335-8. 9054237 10.1093/oxfordjournals.aje.a009110

[10] Linn S, Schoenbaum SC, Monson RR, Rosner B, Stubblefield PG, Ryan KJ. No association between coffee consumption and adverse outcomes of pregnancy. N Engl J Med 1982;306:141-5. 7054656 10.1056/NEJM198201213060304

[11] Cook DG, Peacock JL, Feyerabend C, Carey IM, Jarvis MJ, Anderson HR, et al. Relation of caffeine intake and blood caffeine concentrations during pregnancy to fetal growth: prospective population based study. BMJ 1996;313:1358-62. 8956700 10.1136/bmj.313.7069.1358 PMC2352908

[12] Committee on Toxicity. COT statement on the reproductive effects of caffeine. London: Committee on Toxicity of Chemicals in Food, Consumer Products and the Environment, 2001. http://cot.food.gov.uk/cotstatements/cotstatementsyrs/cotstatements2001/caffeine

[13] Grosso LM, Rosenberg KD, Belanger K, Saftlas AF, Leaderer B, Bracken MB, et al. Maternal caffeine intake and intrauterine growth retardation. Epidemiology 2001;12:447-55. 11428387 10.1097/00001648-200107000-00015

[14] Clausson B, Granath F, Ekbom A, Lundgren S, Nordmark A, Signorello LB, et al. Effect of caffeine exposure during pregnancy on birth weight and gestational age. Am J Epidemiol 2002;155:429-36. 11867354 10.1093/aje/155.5.429

[15] Bracken MB, Triche EW, Belanger K, Hellenbrand K, Leaderer BP. Association of maternal caffeine consumption with decrements in fetal growth. Am J Epidemiol 2002;155:429-36. 12615610 10.1093/aje/kwf220

[16] Santos IS, Victora CG, Huttly S, Carvalhal JB, Santos IS, Victora CG, et al. Caffeine intake and low birth weight: a population-based case-control study. Am J Epidemiol 1998;147:620-7. 9554600 10.1093/oxfordjournals.aje.a009502

[17] Weathersbee PS, Olsen LK, Lodge JR. Caffeine and pregnancy-retrospective survey. Postgrad Med 1977;62:64-5. 896615 10.1080/00325481.1977.11714605

[18] Srisuphan WBM. Caffeine consumption during pregnancy and association with late spontaneous abortion. Am J Obstet Gynecol 1986;154:14-20. 3946486 10.1016/0002-9378(86)90385-6

[19] Goldstein A, Warren R. Passage of caffeine into human gonadal and fetal tissue. Biochem Pharmacol 1962;11:166-8. 13899873 10.1016/0006-2952(62)90106-5

[20] Kirkinen P, Jouppila P, Koivula A, Vuori J, Puukka M. The effect of caffeine on placental and fetal blood flow in human pregnancy. Am J Obstet Gynecol 1983;147:939-42. 6650631 10.1016/0002-9378(83)90250-8

[21] Aldridge A, Aranda JV, Neims AH. Caffeine metabolism in the newborn. Clin Pharmacol Ther 1979;25:447-53. 428190 10.1002/cpt1979254447

[22] Rasmussen BB, Brix TH, Kyvik KO, Brøsen K. The interindividual differences in the 3-demethylation of caffeine alias CYP1A2 is determined by both genetic and environmental factors. Pharmacogenetics 2002;12:473-8. 12172216 10.1097/00008571-200208000-00008

[23] Kotake AN, Schoeller DA, Lambert GH, Baker AL, Schaffer DD, Josephs H. The caffeine CO2 breath test: dose response and route of N-demethylation in smokers and nonsmokers. Clin Pharmacol Ther 1982;32:261-9. 6807598 10.1038/clpt.1982.157

[24] Kalow W, Tang BK. Use of caffeine metabolite ratios to explore CYP1A2 and xanthine oxidase activities. Clin Pharmacol Ther 1991;50:508-19. 1934864 10.1038/clpt.1991.176

[25] Grosso LM, Triche EW, Belanger K, Benowitz NL, Holford TR, Bracken MB. Caffeine metabolites in umbilical cord blood, cytochrome P-450 1A2 activity, and intrauterine growth restriction. Am J Epidemiol 2006;163:1035-41. 16641310 10.1093/aje/kwj125

[26] Boylan SM, Cade JE, Kirk SFL, Greenwood DC, White KLM, Shires S, et al. Assessing caffeine exposure in pregnant women. Br J Nutr 2008, Online publication 10.1017/S0007114508939842. 10.1017/S0007114508939842 18331663

[27] Directorate MFS. Survey of caffeine and other methylxanthines in energy drinks and other caffeine-containing products (updated). Food Surveillance Information Sheet 1998:144.

[28] Butler MA, Lang NP, Young JF, Caporaso NE, Vineis P, Hayes RB, et al. Determination of Cyp1a2 and Nat2 phenotypes in human-populations by analysis of caffeine urinary metabolites. Pharmacogenetics 1992;2:116-27. 1306111 10.1097/00008571-199206000-00003

[29] Binnie V, McHugh S, Macpherson L, Borland B, Moir K, Malik K. The validation of self-reported smoking status by analysing cotinine levels in stimulated and unstimulated saliva, serum and urine. Oral Diseases 2004;10:287-93. 15315646 10.1111/j.1601-0825.2004.01018.x

[30] Gardosi J. Customised fetal growth standards: rationale and clinical application. Semin Perinatol 2004;28:33-40. 15058900 10.1053/j.semperi.2003.12.002

[31] Brown MA, Lindheimer MD, de Swiet M, Van Assche A, Moutquin JM. The classification and diagnosis of the hypertensive disorders of pregnancy: Statement from the International Society for the Study of Hypertension in Pregnancy (ISSHP). Hypertension in Pregnancy 2001;20:IX-XIV. 12044323 10.1081/PRG-100104165

[32] Stata statistical software: Release 10. College station, TX: Stata Corporation, 2007.

[33] Weng X, Odouli R, Li DK. Maternal caffeine consumption during pregnancy and the risk of miscarriage: a prospective cohort study. Am J Obstet Gynecol 2008;198:279 e1-8. 18221932 10.1016/j.ajog.2007.10.803

[34] Rosenberg L, Mitchell AA, Shapiro S, Slone D. Selected birth defects in relation to caffeine-containing beverages. JAMA 1982;247:1429-32. 7035697

[35] Organisation of Teratology Information Specialists. Caffeine and pregnancy. December, 2006. www.otispregnancy.org

[36] Bracken MB, Triche E, Grosso L, Hellenbrand K, Belanger K, Leaderer, et al. Heterogeneity in assessing self-reports of caffeine exposure: implications for studies of health effects. Epidemiology 2002;13:165-71. 11880757 10.1097/00001648-200203000-00011

[37] Bech BH, Obel C, Henriksen TB, Olsen J. Effect of reducing caffeine intake on birth weight and length of gestation: randomised controlled trial. BMJ 2007;334:409-12. 17259189 10.1136/bmj.39062.520648.BE PMC1804137

[38] Bicalho GG, Barros Filho Ade A. [Birthweight and caffeine consumption]. Revista de Saude Publica 2002;36:180-7. 12045799 10.1590/s0034-89102002000200010

[39] Klebanoff MA, Levine RJ, Dersimonian R, Clemens JD, Wilkins DG. Serum caffeine and paraxanthine as markers for reported caffeine intake in pregnancy. Ann Epidemiol 1998;8:107-11. 9491935 10.1016/s1047-2797(97)00125-7

[40] Klebanoff MA, Levine RJ, Clemens JD, Wilkins DG. Maternal serum caffeine metabolites and small-for-gestational age birth. Am J Epidemiol 2002;155:32-7. 11772782 10.1093/aje/155.1.32

[41] Klebanoff MA, Levine RJ, DerSimonian R, Clemens JD, Wilkins DG. Maternal serum paraxanthine, a caffeine metabolite, and the risk of spontaneous abortion. N Engl J Med 1999;341:1639-44. 10572151 10.1056/NEJM199911253412202

